# Use of Glomerular CD68+ Cells as a Surrogate Marker for Endocapillary Hypercellularity in Lupus Nephritis

**DOI:** 10.1016/j.ekir.2021.12.030

**Published:** 2022-01-11

**Authors:** Elisabeth M.J. Bos, Shirish R. Sangle, Suzanne Wilhelmus, Ron Wolterbeek, Natasha Jordan, David D’Cruz, David Isenberg, H. Terence Cook, Jan A. Bruijn, Ingeborg M. Bajema

**Affiliations:** 1 Department of Pathology, Leiden University Medical Center, Leiden, The Netherlands; 2 Louise Coote Lupus Unit, Department of Rheumatology, Guy’s Hospital, London, UK; 3 Pathan B.V., Laboratory for Pathology, Rotterdam, The Netherlands; 4 Department of Medical Statistics, Leiden University Medical Center, Leiden, The Netherlands; 5 Department of Rheumatology, Addenbrooke’s Hospital, Cambridge, UK; 6 Centre for Rheumatology, Division of Medicine, University College London, London, UK; 7 Centre for Inflammatory Diseases, Department of Immunology and Inflammation, Imperial College, London, UK

**Keywords:** activity index, endocapillary hypercellularity, glomerular CD68+ cells, lupus nephritis

## Abstract

**Introduction:**

Lupus nephritis (LN) class III or IV is strongly related to patient mortality and morbidity. The interobserver agreement of endocapillary hypercellularity by routine light microscopy, one of the most important lesions determining whether class III or IV is present, is moderate. In IgA nephropathy (IgAN), the presence of glomerular CD68+ cells was found to be a good surrogate marker for endocapillary hypercellularity. We investigated whether the presence of glomerular CD68+ cells could serve as a surrogate marker for endocapillary hypercellularity as well in LN.

**Methods:**

A total of 92 LN biopsies were scored for the number of glomerular CD68+ cells using CD68 staining, including endocapillary hypercellularity and the activity index (AI). A new AI was calculated in which CD68+ cells replaced endocapillary hypercellularity. Clinical parameters were obtained from time of biopsy, 1 year after, and 2 years after.

**Results:**

The number of glomerular CD68+ cells significantly correlated with endocapillary hypercellularity. A cutoff value of 7 for the maximum number of CD68+ cells within 1 glomerulus in a biopsy yielded a sensitivity of 88% and a specificity of 67% for the presence of endocapillary hypercellularity. Both endocapillary hypercellularity and CD68+ cells correlated with renal function during follow-up. The current and the new AI correlated equally well with the clinical outcome.

**Conclusion:**

In LN, CD68+ cells can be used as a surrogate marker for endocapillary hypercellularity.

## Graphical abstract

**Figure:**
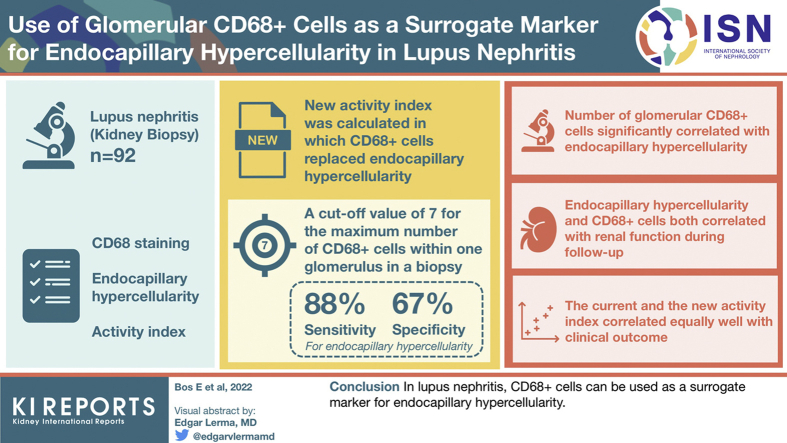

Systemic lupus erythematosus is a highly heterogeneous autoimmune multisystem disease.^1^ LN, a manifestation that occurs in up to 60% of patients with systemic lupus erythematosus, is associated with increased mortality and morbidity. Patients with class III/IV LN have higher mortality and morbidity rates than those with other classes,^2^^,^^3^ and they receive more aggressive immunosuppressants that can have a huge impact on their quality of life.^4^^,^^5^ In class III and IV LN, subendothelial immune deposits elicit influx of inflammatory cells and consequently lead to lesions of which “endocapillary hypercellularity” is the most important. Endocapillary hypercellularity in a single glomerulus is enough to classify the biopsy as class III, which emphasizes the importance of recognizing this lesion.^6^ Unfortunately, the interobserver agreement in recognizing endocapillary hypercellularity is moderate.^7^

It was suggested previously that problems with the definition of endocapillary hypercellularity may be critical to interobserver variation. Therefore, the definition was recently modified in the revised ISN/Renal Pathology Society classification, in which it was stated that influx of inflammatory cells is the most important component of endocapillary hypercellularity and that if an intracapillary lesion has no inflammatory cells but only consists of endothelial swelling, this lesion should not be regarded as endocapillary hypercellularity.^6^ Of all inflammatory cells contributing to endocapillary hypercellularity, monocytes are the most interesting, in particular in relation to the development of the lesion. Monocytes were found to constitute most of the inflammatory cells in the glomeruli of patients with LN affected by class III/IV.^8^ Moreover, monocytes were found to be present in similar hypercellular lesions in other renal diseases, such as IgA nephropathy.^9^

In various renal diseases, the role of monocytes as part of the spectrum of CD68-positive cells was investigated in detail. In IgAN, the presence of CD68+ cells correlated with the occurrence of endocapillary hypercellularity.^10^ In IgAN, there was a similar issue with interobserver variation in relation to this lesion as in LN. Therefore, a study was performed which hypothesized that endocapillary hypercellularity in IgAN mainly reflects glomerular inflammation and that the presence of CD68+ cells would be a more robust marker to determine the E-score. CD68+ cells as a surrogate marker for endocapillary hypercellularity were found to be helpful in decreasing interobserver variation: in IgAN, it was found that if in any glomerulus in a biopsy ≥7 CD68+ cells were present as determined by immunohistochemistry, endocapillary hypercellularity was most likely present, with a sensitivity of 94.1% and a specificity of 71%.^10^ Therefore, in this study, we investigated whether a similar approach could be helpful to recognize endocapillary hypercellularity in LN. We investigated whether CD68 positivity could be used as a surrogate marker for endocapillary hypercellularity in LN.

## Methods

### Study Population

From July 2010 to January 2012, a total of 164 patients with LN were recruited from 2 designated tertiary referral clinical centers. Inclusion criteria were as follows: a definite diagnosis of systemic lupus erythematosus in accordance with the American College of Rheumatology revised classification criteria,^11^ biopsy proven LN, and written informed consent. All study work was conducted in accordance with the requirements of the Helsinki Declaration, and the study was approved by the Outer South East London and the London City Road and Hampstead Research Ethics Committees.

### Renal Histology

Paraffin-embedded renal biopsy tissue was available from 126 patients with LN recruited for this study. Biopsies were traced back to the time of the patients’ original diagnosis (*n =* 107) or if this was not possible, biopsies taken at the onset of a new nephritis flare before induction immunosuppression was commenced were obtained (*n =* 19). In total, 92 cases were included in the study. There were 24 cases that were excluded because the biopsy contained ≤5 glomeruli, 2 cases had inadequate tissue, in 1 case all glomeruli were globally sclerosed (class VI), and for 7 patients insufficient clinical data were available. The classification of a biopsy was recorded from the original pathology report. If a biopsy was classified as either class III + V or class IV + V, it was regarded as class III and IV for analysis.

Endocapillary hypercellularity was re-evaluated blindly by a nephropathologist who was unaware of the original classification and CD68+ cell score, using 1 slide per patient. The presence of endocapillary hypercellularity was scored for each glomerulus. The definition of endocapillary hypercellularity used was as follows: “Endocapillary hypercellularity due to increased number of endothelial cells and infiltrating inflammatory cells, and causing narrowing of the glomerular capillary lumina,’ according to the definition used in the recent recommendations for LN.”^6^ E continuous represents the number of glomeruli in a biopsy with endocapillary hypercellularity in relation to the total number of viable glomeruli.

### Immunohistochemistry

Immunohistochemical staining was performed for CD68. Slides were deparaffinized and subjected to antigen retrieval (Tris/EDTA buffer). After blocking endogenous peroxidase, the sections were incubated with mouse antihuman CD68 (KP-1; Dako, Glostrup, Denmark) for 1 hour. Sections were then counterstained with hematoxylin. Once mounted and dried, the slides were scanned and the number of CD68+ cells in each glomerulus was counted (viewer software: 3DHISTECH Panoramic viewer or Philips Digital Pathology Solution). A maximum CD68+ score was determined for each biopsy, which consisted of the number of CD68+ cells present in a single glomerulus with the highest number of CD68+ cells of that biopsy. The fraction of glomeruli with ≥7 CD68+ cells in a biopsy was determined as based on a receiver operating characteristic analysis (see subsequent texts); this variable was called CD68 continuous.

### Activity Index

All biopsies were scored using the modified National Institutes of Health AI.^6^ To determine whether CD68+ cells are a robust surrogate marker for endocapillary hypercellularity in the context of the AI, a new AI was calculated in which CD68+ cells replace endocapillary hypercellularity. This was done by determining the percentage of glomeruli in which ≥7 CD68+ cells were found. In accordance with the scoring system for parameters of the current AI, a score between 0 and 3 based on the percentage of glomeruli in which ≥7 CD68+ cells were found was given: 0 if none of the glomeruli had ≥7 CD68+ cells, 1 if ≥7 CD68+ cells were present in up to 25% of glomeruli, 2 if ≥7 CD68+ cells were present in 25% to 50% of glomeruli, and 3 if ≥7 CD68+ cells were present in >50% of glomeruli. Next, scores obtained for CD68 positive cells were replaced by scores originally obtained for endocapillary hypercellularity. This new score was called AI CD68. Both the modified AI and the AI CD68 were correlated with clinical data, to establish whether replacing endocapillary hypercellularity by CD68+ cells in the AI would not lead to a loss of correlation with clinical parameters.

### Clinical Variables

We retrospectively collected the clinical data at time of renal biopsy and 1 year and 2 years after the biopsy was taken. We obtained data on sex, age of diagnosis, ancestry, estimated glomerular filtration rate (eGFR), urine protein-to-creatinine ratio, therapy, and whether a renal flare occurred during these 2 years.

### Statistical Analysis

Statistical analysis was performed using SPSS statistics 23.0 (IBM, Armonk, NY). The Kolmogorov-Smirnov test was used to determine whether data were normally distributed. Normally distributed data were presented as mean and SD and analyzed using one-way analysis of variance for categorical data. Continuous variables were correlated using Pearson’s correlation. To test whether correlated Pearson’s rs differed significantly from each other, Hotelling’s T was used.^12^ Non-normally distributed data were presented as median and interquartile range, associations with categorical variables were determined using a Kruskal–Wallis test and Mann–Whitney *U* test, and for continuous variables, this was done using a Spearman correlation test. For non-normally distributed paired data, Wilcoxon’s test and Friedman’s test were used. A cutoff value of the maximum CD68+ score was established using receiver operating characteristic curve analysis. Statistical significance level was set at 0.05.

## Results

**Figure 1:**
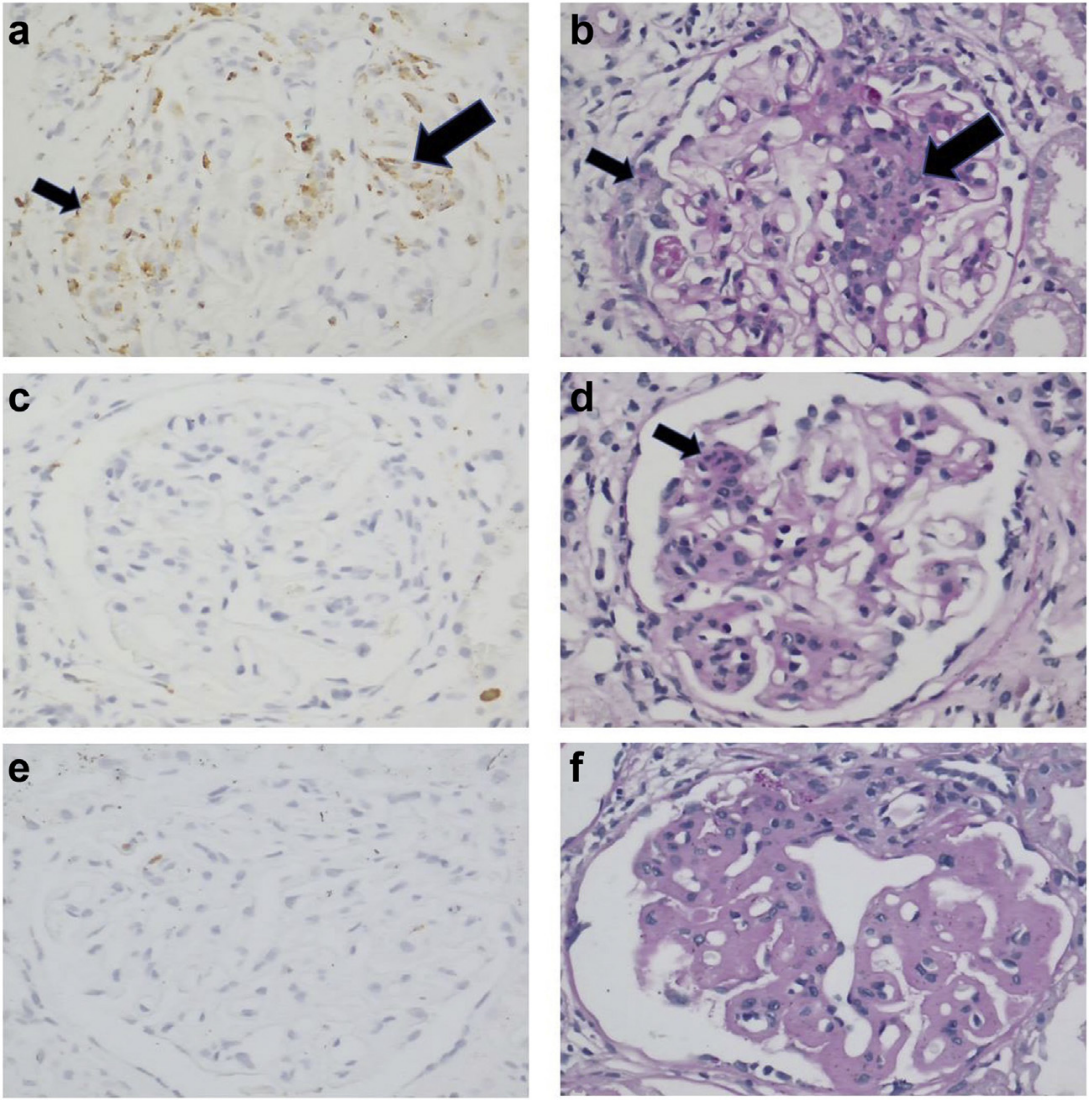
CD68-positive cells in lupus nephritis in relation to histologic findings. (a) LN class IV, glomerulus with abundance of CD68-positive cells. (b) Same glomerulus as found in a; CD68-positive cells are present in areas with endocapillary hypercellularity (big arrows in a and b) but also in extracapillary proliferation (small arrows in a and b). (c) Absence of glomerular CD68-positive cells whereas a small lesion with endocapillary hypercellularity (arrow) is present (d) revealing sampling error may give rise to inconsistent results. (e) Virtual absence of CD68-positive cells in a glomerulus with many wire loops, consistent with class IV but in the absence of endocapillary hypercellularity.

Baseline characteristics are described in Table 1. According to the protocol, the treatment was based on the findings of the kidney biopsy. Therefore, no LN treatment was given before the biopsy. Examples of histology and immunohistochemistry findings are found in Figure 1a-e.

**Table 1:** Baseline characteristics

Characteristics	All patients (*N =* 92)
Female, *n* (%)	76 (82.6)
Age at diagnosis, mean (SD)	26.2 (10.7)
Ethnicity, *n* (%)	
African	7 (7.6)
Afro-Caribbean	28 (30.4)
Asian	21 (22.8)
Caucasian	35 (38.0)
Other	1 (1.1)
Class, *n* (%)	
Class I	6 (6.5)
Class II	8 (8.7)
Class III	28 (30.4)
Class IV	45 (48.9)
Class V	5 (5.4)
Clinical parameters	
eGFR (ml/min per 1.73 m^2^), *n =* 69, median (IQR)	80 (58–106)
uPCR (mg/mmol), *n =* 72, median (IQR)	189 (95–437)
Medication, *n* (%)	
Prednisolone	74 (80.4)
Mycophenolate mofetil	56 (60.9)
Azathioprine	18 (19.6)
Hydroxychloroquine	24 (26.1)

eGFR, estimated glomerular filtration rate; IQR, interquartile range; LN, lupus nephritis; uPCR, urine protein-to-creatinine ratio.

Treatment for LN was initiated after the renal biopsy.

### Renal Histology

The median number of glomeruli per biopsy was 22 (interquartile range 13–33). Endocapillary hypercellularity was found in 74 biopsies (80.4%). The median E continuous in the total cohort was 0.31 (interquartile range 0.07–0.50). The median of the maximum CD68+ score in the whole cohort was 17 (interquartile range 7–33).

**Figure 2:**
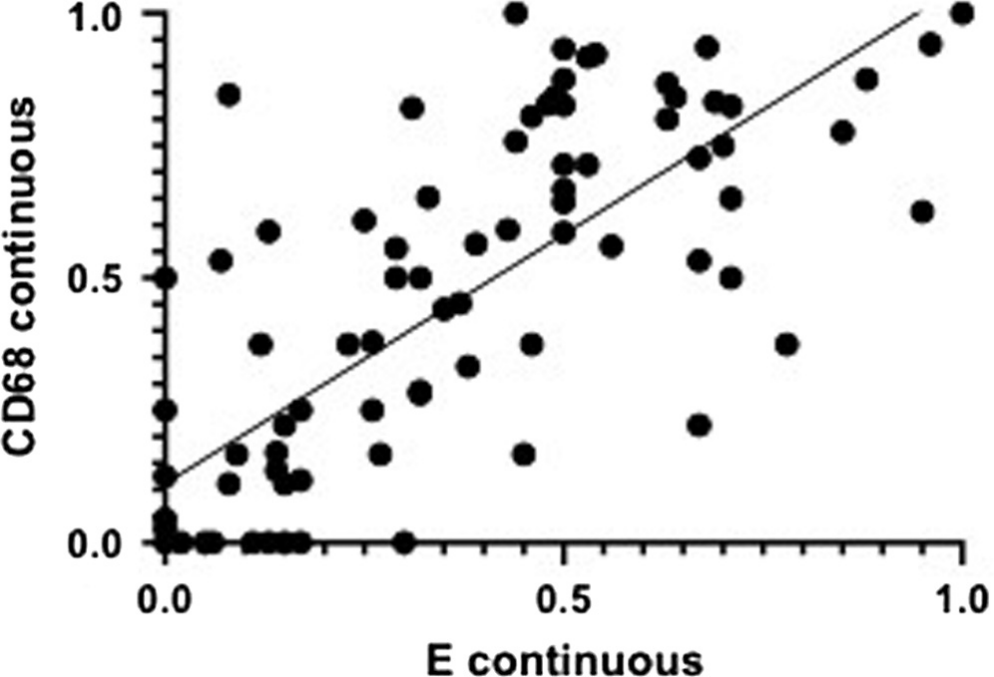
E continuous and CD68 continuous. ρ = 0.784; *P* < 0.001. The fraction of glomeruli with endocapillary hypercellularity and ≥7 CD68+ cells in a biopsy, each dot represents a biopsy.

**Figure 3:**
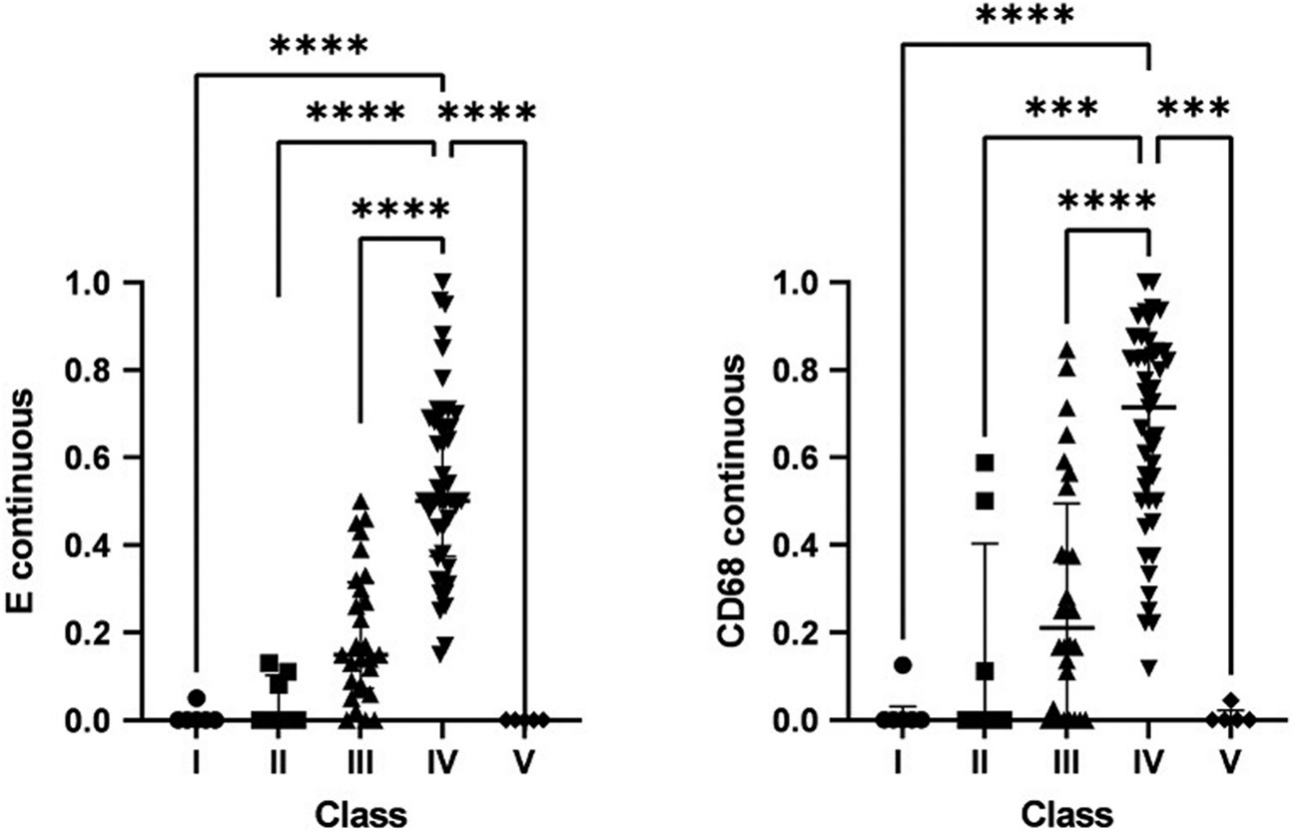
E continuous and CD68 continuous per class. The fraction of glomeruli with endocapillary hypercellularity and ≥7 CD68+ cells in a biopsy compared between classes. Bars represent the median of the group, whiskers the interquartile range. ∗∗∗*P* ≤ 0.001; ∗∗∗∗*P* ≤ 0.0001.

E continuous and CD68 continuous correlated in a statistically significant way (ρ = 0.784; *P* < 0.001) (Figure 2). Both E continuous and CD68 continuous differed significantly between classes (*P* < 0.001 for both) (Figure 3). E continuous and CD68 continuous were highest in class IV patients. The median of the maximum CD68+ cell score was significantly higher (*P* < 0.001) in biopsies with endocapillary hypercellularity than without. Using receiver operating characteristic curve analysis (area under the curve = 0.877), a cutoff value of ≥7 of the maximum CD68 score was determined (Supplementary Figure S1). This generated a sensitivity of 88% and a specificity of 67% (Table 2).

**Table 2:** Endocapillary hypercellularity and maximum CD68 score

Maximum CD68 score	Endocapillary hypercellularity absent	Endocapillary hypercellularity present	Total
Maximum CD68 score < 7	12	9	21
Maximum CD68 score ≥ 7	6	65	71
Total	18	74	92

Number of patients in which endocapillary hypercellularity is either absent or present and in which the maximum CD68 score is <7 or ≥7. Sensitivity = 88%; specificity = 67%.

In 9 patients, endocapillary hypercellularity was scored as present, but the maximum CD68 score was <7. The original classes as stated in the biopsy reports of these patients were class III (7 patients), class I (1 patient), and class II (1 patient). In the detailed scoring process, which was blinded for the original classes, biopsies of the latter 2 patients were scored to have a single glomerulus with an endocapillary lesion. Biopsies originally scored as class III also had only 1 glomerulus with an endocapillary lesion in 4 cases, whereas the other 3 cases had endocapillary hypercellularity lesions in 5 of 29, 3 of 10, and 4 of 23 glomeruli. There were 3 cases in which sampling error accounted for the inconsistency between endocapillary hypercellularity lesion at light microscopy in the absence of a CD68 score of ≥7: in these cases, the glomeruli with the endocapillary hypercellularity lesions were absent in the immunohistochemical staining.

In 6 patients, endocapillary hypercellularity was scored to be absent, but maximum CD68 score was ≥7. Of these patients, 1 was class I, 1 was class II, and 1 was class V. The other 3 patients were classified as class III, likely because of other inflammatory lesions. Two patients were originally classified as classes I and II and who had no endocapillary hypercellularity but did score positive on the presence of neutrophils and karyorrhexis: these 2 biopsies had >7 CD68-positive cells in 5 of 29 and 10 of 17 glomeruli. There was 1 patient originally classified as class V with >7 CD68-positive cells in 1 of 19 glomeruli.

### Clinical Parameters

eGFR was significantly lower in patients with endocapillary hypercellularity at the time of biopsy,1 year after, and 2 years after (*P* < 0.001, *P* = 0.003, and *P* = 0.027, respectively). This was also true for patients with a maximum CD68 score of ≥7 (*P* = 0.005, *P* = 0.028, and *P* = 0.016). Both E continuous and CD68 continuous were correlated with eGFR at time of biopsy (ρ = −0.405; *P* < 0.001 and ρ = −0.295; *P* = 0.008, respectively) and 1 year after biopsy (ρ = −0.259, *P* = 0.022 and ρ = −0.219, *P* = 0.048, respectively). Neither E continuous nor CD68 continuous was significantly correlated to eGFR 2 years after biopsy.

Proteinuria measured by urine protein-to-creatinine ratio did not correlate with the presence of endocapillary hypercellularity, the maximum CD68 score, E continuous, and CD68 continuous at any of the time points.

### Activity Index

The Pearson’s correlation coefficient of the current AI and the AI CD68 that correlated with eGFR was completely comparable, because Hotelling’s T was not significant. This was true for the correlation with eGFR at all 3 time points. Moreover, almost all correlations between both the current AI and the AI CD68 with eGFR were statistically significant (Table 3). Neither the modified AI nor the AI CD68 significantly correlated with urine protein-to-creatinine ratio at any time point.

**Table 3:** Pearson’s correlation between eGFR and modified AI and AI CD68

eGFR	Modified AI	AI CD68	Hotelling’s T
eGFR at time of biopsy	−0.453 (*P* < 0.001)	−0.442 (*P* < 0.001)	−0.508 (*P* = 0.613)
eGFR 1 year after biopsy	−0.218 (*P* = 0.049)	−0.225 (*P* = 0.042)	0.304 (*P* = 0.762)
eGFR 2 years after biopsy	−0.250 (*P* = 0.025)	−0.269 (*P* = 0.015)	0.832 (*P* = 0.408)

AI, activity index; eGFR, estimated glomerular filtration rate.

The correlation between the currently used modified AI and AI CD68, in which the score for endocapillary hypercellularity is replaced by CD68+ cells. Hotelling’s T describes whether the correlated Pearson’s correlations are significantly different, which is not true for any of the time points.

## Discussion

This study reveals that in LN, the presence of glomerular CD68+ cells correlates with the presence of endocapillary hypercellularity. A cutoff value for the maximum CD68+ cell count in a biopsy, which can be helpful in determining whether endocapillary hypercellularity is present in a biopsy, was identified as 7 CD68+ cells, which is comparable to findings in IgAN. We propose that in cases where the pathologist cannot be certain using routine light microscopy whether endocapillary hypercellularity is present, quantifying CD68+ cells can be useful in making this decision. If ≥7 CD68+ cells are found within a glomerulus, it is likely that in that biopsy, endocapillary hypercellularity is present. As a guideline, if <7 cells are present, it could be argued that if an endocapillary hypercellularity lesion is recognized with a relatively high amount of certainty by light microscopy, this overrules the findings by immunohistochemistry.

Neither the sensitivity nor specificity of the cutoff value and the correlation between E continuous and CD68 continuous were perfect. To evaluate this, patients who had endocapillary hypercellularity but a maximum CD68 score of <7 and patients without endocapillary hypercellularity but a maximum CD68 score of ≥7 were studied in more detail. It seems that in biopsies where there is endocapillary hypercellularity in combination with a maximum CD68 score < 7, there is often a very mild class III with, in most cases, only 1 glomerulus revealing endocapillary hypercellularity. The discordance between endocapillary hypercellularity and CD68+ cells was caused by sampling error in some cases. To minimize the risk of sampling error, we recommend the use of consecutive slides to evaluate endocapillary hypercellularity and CD68+ cells and to keep in mind that glomeruli on the edge of the slide may not be present on the other slide.

In our study, the absence of endocapillary hypercellularity in combination with a maximum CD68 score of ≥7 could be due to a class III biopsy based on lesions other than endocapillary hypercellularity, or class I, II, or V with a minority of glomeruli revealing active lesions such as the presence of neutrophils/karyorrhexis or hyaline deposits. Alternatively, CD68 positivity in some patients may be a sign of inflammation that has not progressed to inflammatory lesions, such as endocapillary hypercellularity yet. Therefore, our findings lead to the question whether an overall inflammation score, possibly incorporating immunohistochemistry markers such as CD68, adds to the classification of LN. The international working group of nephropathologists who presented the revised ISN/Renal Pathology Society classification of LN in 2018 addressed this as well. They stated that it should be investigated further if such an overall inflammation score is valuable.^6^ Such an inflammation score could be useful in revealing a continuum of the disease, which may be more precise in describing active inflammation than the current 6 classes of the classification. Moreover, an overall inflammation score may also be helpful as a prognostic tool in subsequent biopsies after treatment, to indicate whether active lesions are present.^13^ In the current study, it was found that by replacing endocapillary hypercellularity in the current AI with a score for glomerular CD68+ cells, virtually no loss of correlation with renal function occurs as compared with the current AI. This indicates that CD68+ cells can be used as a surrogate marker for endocapillary hypercellularity in the context of the AI.

Both the presence of and the fraction of glomeruli with endocapillary hypercellularity and CD68+ cells are associated with worse eGFR at the time of biopsy, 1 year after, and 2 years after. This implies that inflammation, indicated by either the inflammatory lesion endocapillary hypercellularity or the inflammatory CD68+ cells, leads to lower renal function. This inflammation was not correlated with proteinuria. In previous studies, clinical data and outcomes were primarily associated with tubulointerstitial CD68+ cells in patients with LN.^14^

The study by Soares *et al.*^10^ on CD68+ cells in IgAN and this study set out to try to solve the problem of low interobserver agreement of endocapillary hypercellularity, a lesion important in the classification of IgAN and LN, using CD68+ cells as a surrogate marker. In both studies, the maximum CD68 score was used and the cutoff value was identical: ≥7 cells. The similarity of the cutoff value reveals that although these are 2 different diseases, endocapillary hypercellularity lesions may develop from similar mechanism. Sensitivity and specificity were higher in IgAN, and in both studies, specificity was lower than sensitivity. Clinical data were not evaluated by Soares *et al.*^10^ In LN and IgAN, CD68+ cells can be used as a surrogate marker for endocapillary hypercellularity using a similar approach.

The use of CD68+ cells as a surrogate marker for endocapillary hypercellularity has limitations. The specificity is relatively low and sampling errors are possible; for instance, if the area of the glomerulus in which endocapillary hypercellularity is present is not included in the immunohistochemistry slide or vice versa. In addition, an extra staining would need to be performed, leading to higher costs and workload. To validate CD68 as a surrogate marker, the findings of this study should be verified using a validation cohort and more nephropathologists to score endocapillary hypercellularity and CD68+ cells to determine reproducibility. Moreover, in this study, it was not conclusively revealed that quantifying CD68+ cells is more objective and has a higher interobserver agreement compared with endocapillary hypercellularity, although it is likely that this is the case because Soares *et al.*^10^ established a good interobserver agreement for the presence of ≥7 CD68+ cells in a glomerulus.

The results of our study reveal that the presence of CD68-positive cells does not equal the presence of endocapillary hypercellularity; however, there is a strong correlation between the 2 lesions. Using the maximum CD68 score with a cutoff value of 7, CD68+ cells can function as a surrogate marker for endocapillary hypercellularity in LN. Naturally, the presence of CD68 positive cells can reflect lesions other than endocapillary hypercellularity as well. CD68+ cells may be an indicator of overall inflammation within the kidney of patients with LN and could be incorporated into an inflammation score, either in addition to or incorporated in the current AI. A new AI in which CD68+ cells were included correlates with clinical outcome equally well as the current AI, further revealing the importance of CD68+ cells as a marker of inflammation in LN.

## Disclosure

DDC reports receiving consulting fees and speaker honorarium from GlaxoSmithKline. IB reports receiving consulting fees from Aurinia Pharmaceuticals, Boehringer Ingelheim, and SDE Research. All the other authors declared no competing interests.

## Supplementary Material

Supplementary File (PDF)

**Figure S1.** ROC curve. ROC curve of maximum CD68 score to determine whether endocapillary hypercellularity is present.

**STROBE Statement.**
