# Supplementary material for: Use of Glomerular CD68+ Cells as a Surrogate Marker for Endocapillary Hypercellularity in Lupus Nephritis
Source: Kidney Int Rep. 2022 Jan 11;7(4):841–7. doi: 10.1016/j.ekir.2021.12.030 (PMC9039478; doi:10.1016/j.ekir.2021.12.030)
Supplement: Supplementary File (PDF) [file mmc1.pdf]

Supplementary material

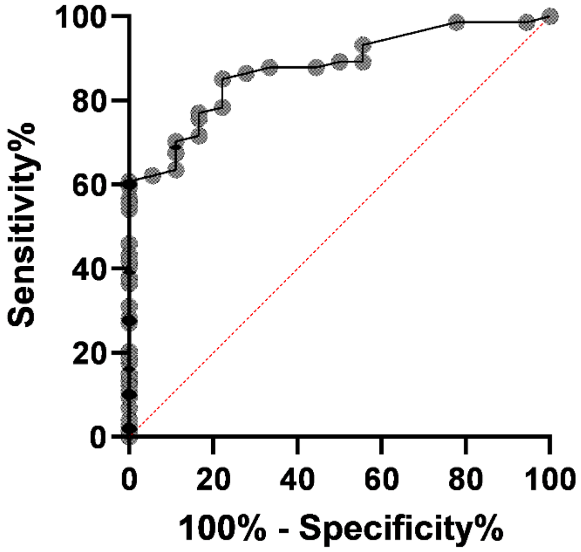

Supplementary Figure 1 ROC curve. ROC curve of maximum CD68 score to determine whether endocapillary hypercellularity is present.

Modified STROBE Statement—checklist of items that should be included in reports of observational studies (Cohort/Cross-sectional and case-control studies)

	Item No	Recommendation
**Title and abstract**	1	(*a*) Indicate the study's design with a commonly used term in the title or the abstract Abstract: second paragraph
		(*b*) Provide in the abstract an informative and balanced summary of what was done and what was found Abstract: first and second paragraph
**Introduction**		
Background/rationale	2	Explain the scientific background and rationale for the investigation being reported Introduction: first, second and third paragraph
Objectives	3	State specific objectives, including any prespecified hypotheses Introduction: last paragraph (final sentences)
**Methods**		
Study design	4	Present key elements of study design early in the paper Methods: subheading 'study design and population'
Setting	5	Describe the setting, locations, and relevant dates, including periods of recruitment, exposure, follow-up, and data collection Methods: subheadings 'study design and population' and 'clinical variables'
Participants	6	(*a*) *Cohort study*—Give the eligibility criteria, and the sources and methods of selection of participants. Describe methods of follow-up Methods: subheadings 'study design and population' and 'clinical variables'<br><br>*Case-control study*—Give the eligibility criteria, and the sources and methods of case ascertainment and control selection. Give the rationale for the choice of cases and controls<br><br>*Cross-sectional study*—Give the eligibility criteria, and the sources and methods of selection of participants
Variables	7	Clearly define all outcomes, exposures, predictors, potential confounders, and effect modifiers. Give diagnostic criteria, if applicable Methods : subheadings 'renal histology', 'immunohistochemistry', 'activity index' and 'clinical variables'
Data sources/ measurement	8*	For each variable of interest, give sources of data and details of methods of assessment (measurement). applicable Methods : subheadings 'renal histology', 'immunohistochemistry', 'activity index' and 'clinical variables'

Bias	9	Describe any efforts to address potential sources of bias Methods: 'renal' histology' (second paragraph)
Study size	10	Explain how the study size was arrived at (if applicable) Methods: sub headings 'study population' and 'renal histology'
Quantitative variables	11	Explain how quantitative variables were handled in the analyses. If applicable, describe which groupings were chosen and why Methods : 'renal histology', 'immunohistochemistry' and 'activity index'
Statistical methods	12	(*a*) Describe all statistical methods, including those used to control for confounding Methods: statistical analysis
		(*b*) Describe any methods used to examine subgroups and interactions Methods: statistical analysis
		(*c*) Explain how missing data were addressed Methods: statistical analysis.
		(*d*) *Cohort study*—If applicable, explain how loss to follow-up was addressed n/a<br>*Case-control study*—If applicable, explain how matching of cases and controls was addressed<br>*Cross-sectional study*—If applicable, describe analytical methods taking account of sampling strategy
		(*e*) Describe any sensitivity analyses n/a
**Results**		
Participants	13*	(a) Report numbers of individuals at each stage of study—eg numbers potentially eligible, examined for eligibility, confirmed eligible, included in the study, completing follow-up, and analyzed Described thoroughly in methods: Methods: sub headings 'study population' and 'renal histology'
		(c) **Use of a flow diagram described in text**
Descriptive data	14*	(a) Give characteristics of study participants (eg demographic, clinical, social) and information on exposures and potential confounders Results: first paragraph and Table 1
		(b) Indicate number of participants with missing data for each variable of interest Table 1 (applies to clinical parameters)
		(c) *Cohort study*—Summarise follow-up time (eg, average and total amount) two years for all study participants
Outcome data	15*	*Cohort study*—Report numbers of outcome events or summary measures over time Results: sub heading 'clinical parameters'

		Case-control study—Report numbers in each exposure category, or summary measures of exposure
		Cross-sectional study—Report numbers of outcome events or summary measures
Main results	16	(*a*) Give unadjusted estimates and, if applicable, confounder-adjusted estimates and their precision (eg, 95% confidence interval). Make clear which confounders were adjusted for and why they were included Results: sub headings 'renal histology', 'clinical parameters' and 'activity index'
Other analyses	17	Report other analyses done—eg analyses of subgroups and interactions, and sensitivity analyses Results: sub heading 'renal histology': third and fourth paragraph
**Discussion**		
Key results	18	Summarise key results with reference to study objectives Discussion: first and seventh (last) paragraph
Limitations	19	Discuss limitations of the study, taking into account sources of potential bias or imprecision. Discuss both direction and magnitude of any potential bias Discussion : Sixth paragraph
Interpretation	20	Give a cautious overall interpretation of results considering objectives, limitations, multiplicity of analyses, results from similar studies, and other relevant evidence Discussion: all paragraphs
Generalisability	21	Discuss the generalisability (external validity) of the study results Discussion: seventh (last) paragraph

*Give information separately for cases and controls in case-control studies and, if applicable, for exposed and unexposed groups in cohort and cross-sectional studies.

Note: An Explanation and Elaboration article discusses each checklist item and gives methodological background and published examples of transparent reporting. The STROBE checklist is best used in conjunction with this article (freely available on the Web sites of PLoS Medicine at http://www.plosmedicine.org/, Annals of Internal Medicine at http://www.annals.org/, and Epidemiology at http://www.epidem.com/). Information on the STROBE Initiative is available at www.strobe-statement.org.
